# Developments in Carbohydrate-Based Cancer Therapeutics

**DOI:** 10.3390/ph12020084

**Published:** 2019-06-04

**Authors:** Farzana Hossain, Peter R. Andreana

**Affiliations:** Department of Chemistry and Biochemistry, University of Toledo, Toledo, OH 43606, USA; Farzana.Hossain@rockets.utoledo.edu

**Keywords:** cancer treatment, carbohydrate antigens, carbohydrate-based antitumor vaccines, warburg effect, iminosugar, cancer diagnosis

## Abstract

Cancer cells of diverse origins express extracellular tumor-specific carbohydrate antigens (TACAs) because of aberrant glycosylation. Overexpressed TACAs on the surface of tumor cells are considered biomarkers for cancer detection and have always been prioritized for the development of novel carbohydrate-based anti-cancer vaccines. In recent years, progress has been made in developing synthetic, carbohydrate-based antitumor vaccines to improve immune responses associated with targeting these specific antigens. Tumor cells also exhaust more energy for proliferation than normal cells, by consuming excessive amounts of glucose via overexpressed sugar binding or transporting receptors located in the cellular membrane. Furthermore, inspired by the Warburg effect, glycoconjugation strategies of anticancer drugs have gained considerable attention from the scientific community. This review highlights a small cohort of recent efforts which have been made in carbohydrate-based cancer treatments, including vaccine design and the development of glycoconjugate prodrugs, glycosidase inhibiting iminosugars, and early cancer diagnosis.

## 1. Introduction

Carbohydrates are the most abundant complex biomolecules, which play pivotal roles in many cellular interactions, such as signaling to other cellular molecules or cell surface receptors [1]. A wide range of monosaccharide and oligosaccharide residues are connected by glyosidic linkages to form essential glycoconjugates, including glycoproteins, glycolipids, and glycosylated natural products. Furthermore, the biosynthesis of those glycans is controlled by several enzymes, so any deviation in the structure of cell surface glycans enables them to encode information essential for disease progression [2]. Therefore, carbohydrates, which can induce glycan-mediated interactions, are targeted as pharmaceutical therapeutic agents aimed at treating various pathological diseases. 

Historically, many naturally isolated carbohydrates were initially used for the development of cancer diagnostic tools. As time elapsed, scientists began to embrace synthesizing carbohydrates for a number of reasons, including the often difficult and lengthy process of obtaining reasonable quantities of pure compound from natural sources. For example, many research groups have relegated to synthesizing tumor-associated carbohydrate antigens (TACAs), which have been noted by the National Institutes of Health as important biomarkers of cancer prognosis, rather than enduring a cumbersome isolation strategy [3]. In many instances, TACAs alone have been found to be poorly immunogenic, unable to induce a T-cell dependent immune response, which has been noted as critical for cancer therapy [3]. At some point in time, scientists began to conjugate TACAs with T-cell stimulating protein carriers, including keyhole limpet haemocyanin (KLH), tetanus toxoid (TT), bovine serum albumin (BSA), and diphtheria toxin (CRM197) [4]. Initially, the responses of those monovalent vaccines were promising, but with further studies, those protein carriers themselves were found to act as self-immunogenic and suppress antigen-specific immunogenicity [5]. Subsequently, TACAs have been coupled with polysaccharides (zwitterionic polysaccharide, PS A1) [6], Toll-like receptor 2 (TLR2) ligand, Pam_3_CysSerK_4_ [7], and T-cell peptide epitopes [8], among others, to develop partially to fully synthetic, self-adjuvating, multi-component cancer vaccines. Some of those aforementioned vaccines have been able to reach different phases of clinical trials, e.g., a hexavalent vaccine construct, incorporating GM2, globo H, Le^y^, clustered Thomsen nouveau (Tn), clustered Thomsen-Friedenreich (TF), and glycosylated mucin 1 (MUC1) antigens have been used for the treatment of phase II prostate cancer patients [9].

Aside from carbohydrate-based tumor antigens, cancer cells also contain an increased number of glucose transporters (GLUTs) and lectins on their membrane surface, which can transport or bind carbohydrate moieties, respectively. The demand for increased energy in proliferation of cancer cells is met by GLUTs, which allow for an increased uptake of glucose at a higher rate than normal cells—a phenomenon commonly referred to as the “Warburg effect” [10]. This effect has garnered much attention from the community, as many scientists have designed and developed sugar-based targeted drug delivery. Several cytotoxic agents, e.g., glufosfamide, chlorambucil, busulfan, docetaxel, paclitaxel, have been glycoconjugated and found to be less toxic to normal cells than the parent aglycons [11]. Those sugar prodrugs are thought to be cleaved by various intracellular glycosidases. The majority of carbohydrate-based prodrugs are used to improve pharmacokinetic properties, and the site of glycosidase cleavage is typically extracellular, allowing for the release of active drugs. Further research, however, is required to validate the GLUT-mediated cellular entry or GLUT inhibition of those drugs.

The biosynthesis of certain glycans, such as *N*-glycans, by altered glycosylation is also considered a well-known hallmark for cancer progression. Enhanced expression of various glycosyltransferase enzymes, including *N*-acetylglucosaminyltransferase V (e.g., GalNAc-TV, GnT-V, MGAT5), are responsible for an increased number of *N*-glycans in tumor cells [12]. Some imino natural alkaloids (e.g., swainsonine, deoxymannojirimycin, castanospermine) were found to be good inhibitors of specific glycosidases, thereby blocking complete *N*-glycan processing. Numerous iminoalditols and their analogs have been synthesized and inhibitory activity analyzed [13].

Over the past few decades, there have also been enormous strides in development with various sectors of cancer therapeutics, however, patient survival rates are still low when diagnosis is in late stage tumor progression. Only a few plasma tumor markers, such as prostate specific antigen (PSA), cancer antigen 125 (CA125), and alpha-fetoprotein (AFP), have been clinically used for early stage cancer diagnosis in the United States [14]. Most of the plasma tumor antigens are neither sensitive nor specific enough to detect at a very early stage. Recently, some carbohydrate-based non-invasive diagnosis cancer tools, such as metabolic oligosaccharide engineering (MOE) imaging technology, lectin binding, and glycan micro-arrays, have been used to screen tumors [15].

Although a large amount of data is available, this review will mainly focus on glycoconjugate therapeutics, which have been recently used for cancer treatment and prevention. First, we will discuss the recent carbohydrate-based vaccine developments with improved immune responses, glycoconjugated cytotoxic prodrugs for targeted drug delivery, glucosidase inhibiting iminosugars, and finally early cancer detection.

## 2. Immune Therapy with Carbohydrate-Based Vaccines

### 2.1. TACAs and Their Immune Response

Oligosaccharides, which coat the plasma membrane, are linked to proteins or lipids through the machinery of glycosyltransferases. Usually, the sugar moieties of glycolipids are attached to a ceramide chain, and for glycoproteins those moieties are linked to the peptide backbone of proteins via an *N*-linkage (linked to NH residue of arginine or lysine or an *O*-linkage (linked to OH residue of serine or threonine. As oligosaccharides are embedded on the cell surface, they become a point of initial contact for many cellular interactions, including the biological transmission of signals, adhesion of lections, release of cytotoxins, and elicitation of antibodies [16]. However, aberrant or modified glycosylation patterns on cancer cell surfaces are typically associated with up- or down-regulation of many glycosyltransferases. For example, in vitro studies reveal that elevations of serum glycosidases, e.g., *β*-*N*-acetylglucosaminidase and *β*-glucuronidase, have been found to occur in different cancer cells [17]. Resultant abnormal glycans are overexpressed on many carcinomas, including those in biopsies of cancer patients. 

TACAs are distinctly marked in large number of tumors, but not on normal cells. They promote tumor cell invasion, cause metastasis, and are immunogenic, making them unique targets for cancer vaccine design and development. TACAs are divided into two classes [18]. One is protein linked, including the Tn, Sialyl-Tn (STn) and TF antigens. These antigens are conjugated to the -OH group of serine and threonine residues, and are the result of truncated glycosylation and premature sialylation of protein (e.g., Mucin protein). The mutation in molecular chaperone, Cosmc, is responsible for the lack of cellular *β*-3-galactosyltransferase (T-synthase), which promotes Tn-antigen (*α*-GalNAc-Thr/Ser) formation [19,20]. Furthermore, the blood group precursors, Tn, STn, and TF-antigens, contribute to form an array of antigens on heavily glycosylated mucin proteins, such as MUC1. Another classification of TACAs is glycolipid-based. This classification contains the gangliosides GM2, GD2, GD3, fucosyl-GM1, Globo-H, and Lewis^y^ (Le^y^), which all contain a lipidated reducing end [3]. The glycosphingolipids GM2, GD2, and GD3 are involved in human melanomas and Lewis antigens, such as sialyl Lewis (SLe^a^), SLe^x^, SLe^x^-Le^x^, are identified human tumor-associated antigens. However, difficulties in the isolation of those antigens from natural sources, due to the heterogeneity of sugars on the cell-surface of tumor cells, complicates vaccine design and development. Therefore, many carbohydrate-based research groups have been resolved to synthesizing homogeneous sugar antigen constructs, and synthetic efforts are underway to facilitate and advance relevant methods, including one-pot synthesis [21] and automated oligosaccharide synthesis [22], to expand the chemists’ reagent repertoire for oligomer assembly. 

**Figure 1:**
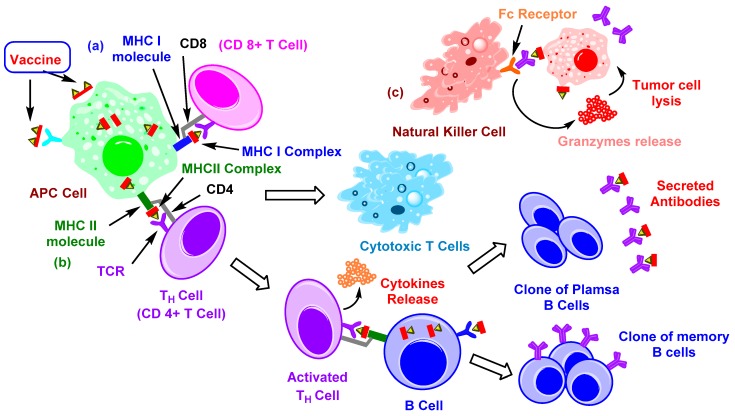
Illustration of immune response to cancer cells. (**a**) Vaccine constructs containing specific antigen(s) get internalized inside antigen-presenting cells (APCs) via endocytosis or binding with specific receptors. While inside APCs, immunogens get proteolyzed by immune proteasomes and divided into several peptide fragments containing antigen(s). If those fragments get loaded onto MHC I then they form MHC I complexes. The resulting complex is transported to the surface so that it can be recognized by CD8+ T-cells. Activated T-cells proliferate to give cytotoxic T-cells [23,27], (**b**) fragments binding with MHC II molecules result in an MHC II complex, which is then transported to the cell surface, activating CD4+ T-cells. Resulting activated cells can further activate B-cells, which present similar antigenic fragments with MHC II. Activated B-cells differentiate into clones of plasma and memory B-cells [23,27], (**c**) antibody-dependent cellular cytotoxicity (ADCC) occurs when IgG antibodies bind with tumor cells, presenting the target specific antigen(s), then Fc receptors of natural killer (NK) cells can recognize them and release granzymes (perforin, proteases, etc.), which causes lysis of tumor cells [28].

The feature which hampers the development of carbohydrate-based vaccines is their T-cell independent nature. Furthermore, TACAs are considered “self-antigens” and elicit B-cell dependent specific IgM antibodies (possibly IgG3), with no memory T-cell response. The antibodies against carbohydrate antigens are known to exhibit complement-dependent cytotoxicity (CDC), antibody-dependent cellular cytotoxicity (ADCC), and interfere with receptor-mediated signaling to combat tumor cells [23]. After the binding of carbohydrate antigens with the Ig receptors of B-lymphocytes, they can induce cross-linking with Ig proteins, thus activating B-cells and producing low affinity IgM antibodies [24]. However, to get a high-affinity IgG response via class switching, B-cells are required to communicate with T-helper or CD4+ cells. On the other hand, participation of the antigen-presenting cells (APCs) is necessary for activation of the T-cells (Figure 1). APCs can capture, internalize, and proteolytically cleave the protein antigens into peptide fragments (~12–15 amino acids long). Those fragments, containing specific antigens, can be presented on the surface of the APC cells and form a complex with the major histocompatibility complex (MHC) class II molecules. Afterward, APC cells can migrate to lymphoid organs, and MHC class II-peptide complex can interact with T-cell receptors of naïve T-lymphocytes—they then become activated [7]. Furthermore, cluster of differentiation 40 ligand (CD40L) receptors of activated T-helper cells bind with the CD40 on B-cells, resulting in cytokine signaling by the T-cell. The combination of binding to CD40 and cytokine production can stimulate B-cells to proliferate and differentiate into plasma and memory B-cells. The plasma B-cell can secrete antibodies, which can bind with specific surface antigens on cancer cells. Similarly, long-lived memory B-cells rapidly secrete high affinity antibodies (IgG) on subsequent exposure to antigens [18]. Furthermore, CD8+ T-cells are also able to recognize glycoconjugated TACAs (e.g., Tn, TF) with peptides (e.g., MUC1), which have optimal binding affinity for MHC I molecules [25,26]. 

### 2.2. Carrier-Based Carbohydrate Conjugates

**Figure 2:**
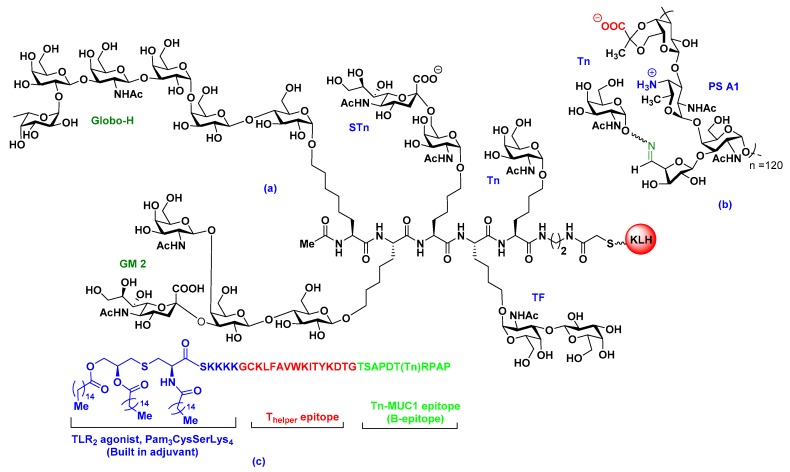
Recent development of tumor-associated carbohydrate antigen (TACA) vaccines. (**a**) Multicomponent vaccine containing different TACAs [23,31], (**b**) entirely carbohydrate-based semi-synthetic vaccine with naturally occurring zwitterionic polysaccharide [34], (**c**) fully synthetic carbohydrate vaccine containing Pam_3_CysSerLys_4_, T-helper epitope, and Tn-MUC1 epitope [7,35].

To stimulate additional T-cell responses, early attempts to develop carbohydrate–protein conjugate vaccines involved the conjugation of isolated TACAs with a carrier protein, such as keyhole limpet hemocyanin (KLH), bovine serum albumin (BSA), diphtheria toxoid (DT), tetanus toxoid (TT), ovalbumin, human serum albumin (HSA), meningococcal outer membrane protein complex (OMPC), *Hemophilus influenzae* protein D, or *Pseudomonas aeruginosa* exotoxin A (rEPA) [16,18]. Recently, clinical trials of GD3 ganglioside vaccines and anti-idiotypic monoclonal antibodies, which mimics GD3 gangliosides, were carried out on melanoma patients [29]. The patients were sequentially immunized with BEC2, anti-idiotypic monoclonal antibody vaccine mimicking GD3, followed by GD3-lactone-KLH (GD3-L-KLH), or vice versa. Anti-GD3 antibodies were responsive to the GD3-L-KLH vaccine, but there was a noted poor correlation with previous studies and the result was a low survival outcome [29]. Based on previous immune responses, several ganglioside-KLHs have been synthesized and further clinical studies have been carried out [30]. The results obtained led to the synthesis and structural modifications of TACAs to improve immunogenicity. Over the past number of years, Livingston–Danishefsky research teams made enormous contributions to the carbohydrate-based vaccine development field. They reported on the synthesis of a number of oligosaccharides, glycoconjugates, and TACAs, including Globo-H, Lewis^y^, Lewis^x^, Lewis^b^, KH-1, MUC1, GM2, STn, and Tn, and evaluated, preclinically, the first generation monovalent KLH-conjugate vaccines [16]. Later, they developed some multicomponent vaccines by combining different TACAs on a polypeptide backbone and finally linking it to KLH (Figure 2a), and further clinical trials have been carried out with a collaboration at the Memorial Sloan Kettering Cancer Center (MSKCC) [31,32]. Wong et al. also synthesized Globo H vaccines using several protein carriers, such as keyhole limpet hemocyanion, diphtheria toxoid cross-reactive material CRM197 (DT), tetanus toxoid, and BSA [33]. Among them, the Globo H-diphtheria toxoid (GH-DT) vaccine in the presence of *α*-galactosylceramide C34 adjuvant was able to induce the highest anti-GloboH IgG antibodies for targeting breast cancer cells [33].

The classical method for conjugating TACAs with a protein involves the utilization of a linker moiety. It has been well documented that linkers themselves can be self-immunogenic or even suppress the antibody production against carbohydrate antigens. For example, in 2004, Boons et al. reported that the immunogenicity of the Le^y^ conjugate vaccine was suppressed by a rigid bifunctional cross-linker—cyclohexyl maleimide [23]. The linker produced strong IgM and IgG responses, but when a more flexible 3-(bromoacetamido)-propionate linker was used, lower titers of antibody production against the linker was observed [23]. Recently, a number of linkers, including succinimide esters, *m*-maleimidobenzoyl hydrazide, 4-(4-*N*-maleimidomethyl)cyclohexane-1-carboxyl hydrazide, squaric acid diesters, and *p*-nitrophenol esters, have been utilized, depending on the relatively easily accessible functional groups on the carrier protein and carbohydrate antigen [16]. Other concerns regarding linkers are chain length, water solubility, low yield, and first attachment either to a protein or antigen. Even though protein conjugate vaccines contributed to the initial development of tumor therapeutic vaccines, the immunogenicity of the desired TACA or epitope is now known to be suppressed by inherently immunogenic protein carrier. 

As an alternative to carrier proteins, our group has isolated several zwitterionic polysaccharides (ZPSs), including PS A1 and PS B from anerobic *Bacteroides fragilis* (ATCC 25285/NCTC 9343) and specific type 1 polysaccharide (Sp1) from *Streptococcus pneumoniae* serotype 1. Like some carrier proteins, ZPSs are also known to elicit a CD4+ T-cell dependent immune response and invoke class switching from IgM to IgG [36]. The co-stimulatory molecules CD40 and CD86 or CD80 on the surface of APCs also can be induced by PS A1 [37]. PS A1 is also known to bind with toll like receptor-2 (TLR-2) of dendritic cells, which plays an active role in releasing IL-12 and IFN-γ [38]. 

Our group has synthesized aminooxy Tn, TF, STn, and Globo-H antigens and conjugated them to chemically treated, oxidized PS A1, aiming to develop entirely carbohydrate-based cancer vaccines (Figure 2b) [6,34]. The vaccine constructs were injected into C57BL/6J mice, either in the presence or absence of the TiterMax® Gold and Sigma adjuvant system (SAS)®, which generated antigen specific, highly robust immune responses (IgM and IgG) noted in enzyme-linked immunosorbent assay (ELISA) [6,34]. Antibody responses of Tn-PS A1 from adjuvant-free vaccinated mice sera indicate the possibility of a dual role of PS A1 as both carrier and adjuvant. Further flow cytometry (FACS) data, with TF and STn-PS B vaccines, also indicated antibody binding to TF-laced MCF-7 cells. Recently, our group has synthesized a tetrasaccharide repeating unit of PS A1, with alternative charges on adjacent monosaccharides, and experiments are underway to unlock the mystery surrounding unknown aspects of carbohydrate immunity [39]. 

### 2.3. Fully Synthetic Carbohydrate Vaccines

To avoid immunosuppressive carrier proteins, many self-adjuvating, multicomponent, fully synthetic vaccines have been proposed by a number of research groups. For example, Boons et al. proposed a multicomponent vaccine to elicit both cytotoxic T lymphocytes (CTLs) and antibody-dependent cellular cytotoxicity (ADCC)-mediated humoral immunity [7]. The tripartite vaccine is comprised of the immunoadjuvant Pam_3_CysSK_4_, a peptide T-helper epitope, and an aberrantly glycosylated MUC1 peptide (B-epitope) (Figure 2c) [7]. The TLR2 ligand is known to enhance local inflammation and activate the components of the adaptive immune system. The vaccine containing glycosylated MUC1 was more lytic compared to a non-glycosylated counterpart. The mucin 1 (MUC1) is a transmembrane protein overexpressed in various tumors, like lung, breast, pancreas, kidney, ovary, and colon tumors. The extracellular *N*-terminal domain of MUC1 contains a variable number of 20 amino acid tandem repeat (VNTR) units, like HGVTSAPDTRPAPGSTAPPA. It is aberrantly glycosylated in cancer cells but highly glycosylated in normal cells. Because of this distinguishable characteristic, the National Cancer Institute (NCI) has declared MUC1 as a prioritized cancer antigen among 75 TACAs, therefore many research groups are attempting to develop vaccine constructs utilizing peptide backbones present in VNTR [35]. 

Mice are usually immunized with vaccines in the presence of adjuvants, which is thought to make the vaccine immunogenic enough to ignore the self-tolerance immunogenicity towards TACAs and boost the immune response [40]. Recently, self-adjuvating vaccines, containing the immunogenic antigens as well as the adjuvants, in a single-entity, have begun to draw much attention in the vaccine arena. For example, in 2017, Yin and co-workers reported a fully synthetic self-adjuvating vaccine candidate [41]. This two-component vaccine contains: (i) an invariant natural killer T (iNKT) cell ligand, α-galactosylceramide (*α*GalCer), and (ii) a sialyl Tn (STn). This STn-*α*GalCer vaccine construct showed remarkable efficacy in inducing antibody class switching from IgM to STn-specific IgG (IgG1 and IgG3 subtypes) antibodies [41].

To improve the antigen stability against glycosidases and enhance the in vivo bioavailability, a fully synthetic vaccine has been reported. The vaccine is comprised of four clustered Tn-antigen analogs, an immunostimulant peptide (OvaPADRE), and a cyclopeptide scaffold. This vaccine prototype, thus far, has elicited long-lasting antibodies able to bind Tn expressing MCF-7 human breast cancer cells, and it was observed to produce high titers of IgG1, IgG2a, and IgG3 antibodies [42]. 

## 3. Glycosylation for Specific Anticancer Drug Delivery

### 3.1. Glucose Metabolism in Cancer Cells and Warburg Effects

**Figure 3:**
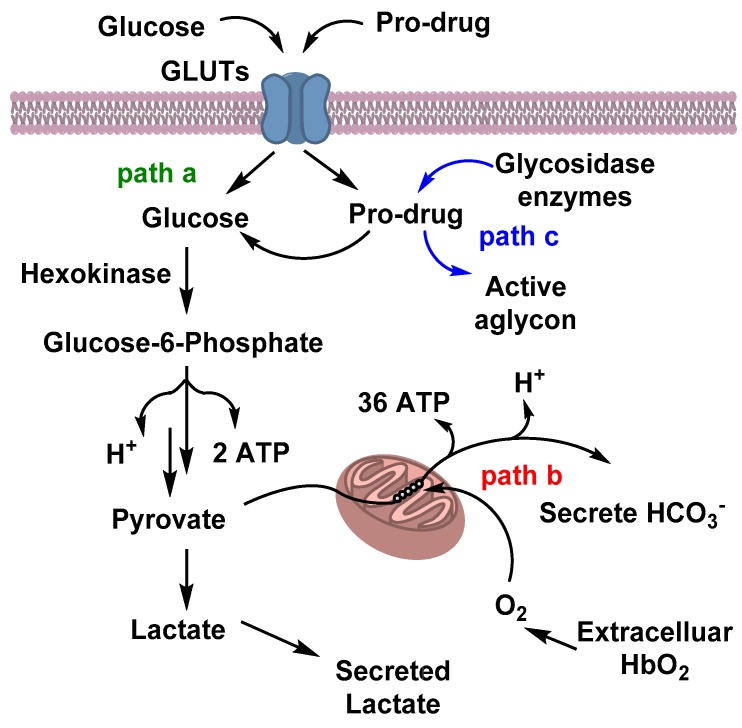
Glucose metabolism and prodrug route inside the cells: Glucose or glyco-conjugated pro drugs get internalized inside the cells via glucose transporters (GLUTs). Glucose metabolism follows either **path a**—anaerobic glycosylation; **path b**—aerobic glycosylation; or **path c**—cleavage of the active drug by a glycosydic enzyme.

Unlike normal cells, many cancer cells consume an abundance of glucose, and have a higher rate of aerobic glycolysis to supply the increased energy required for their rapid proliferation [43]. Tumor cells can alter cellular metabolism during the transition from a normal to abnormal state. Normally, during glucose metabolism or glycolysis, one molecule of glucose is converted into two pyruvates, two molecules of ATP, and two reduced nicotinamide adenine dinucleotide (NADH) molecules. In the presence of oxygen, pyruvate undergoes oxidation to CO_2_ and H_2_O, and generates 36 additional ATPs per glucose (Figure 3), whereas in the absence of oxygen, pyruvate gets reduced to lactic acid [44]. However, in cancer cells, a large amount of glucose can also be converted to lactic acid, regardless of the availability of oxygen. This kind of unusual metabolism opens the windows for many cancer-targeting therapeutics. This tendency of a higher rate of glucose consumption or increased aerobic glycosylation phenomenon, known as the Warburg effect, was first observed by the German scientist Otto Warburg in 1926, and has become an object of significant interest in defeating cancer [10]. The Warburg Effect was later utilized in a clinical study, when the use of a radio labeled glucose-analog, 2-deoxy-2(^18^F)fluoro-D-glucose (^18^F-FDG), was consumed by cancer cells observed via positron emission tomography (PET) analysis. This study is now considered one of the hallmarks in the fight against cancer [45]. Based on this effect, many glycosylated prodrugs have been designed over the past few years with the aim of a targeted delivery of respective active anticancer drugs towards cancer cells, and to improve pharmacokinetics, like water solubility and serum stability [46].

For the transportation of polar molecules, especially sugars, inside cells, some carrier proteins are located on the plasma membrane. There are two types of transporter families: one is facilitated glucose transporters (GLUTs), which facilitates glucose transportation between external and internal plasma membrane via concentration gradient [43]. To date, 14 different kinds of GLUTs (GLUT-1–14) have been classified according to their structure and sequence. Among them, GLUT-1 is the most reported facilitative sugar transporter and is known to be overexpressed in cancerous cells [47]. Another kind of transporter is known as sodium-dependent sugar transporter (SGLT), which requires energy for sugar transport [43]. Many cancer cells have overexpressed glucose transporters (GLUTs) to facilitate a higher glucose consumption than that of normal cells. This observation has opened new windows for targeted chemotherapy with fewer side effects. Many groups have conjugated sugars at different positions of cytotoxic agents and analyzed their ability to target glucose transporters, especially GLUT-1. However, to analyze GLUT-1 receptor mediated transportation of glycan-based paclitaxel prodrugs, the receptor was co-treated with GLUT-1 inhibitors, such as phloretin and phlorizin [48]. Due to the similarity in structures among the GLUTs, it is difficult to determine which one gets targeted by glucose-conjugates. No prodrug co-crystalized with human GLUT-1 is known due to rapid interchangeable conformations, which impede crystallization [49]. However, after cellular uptake, the effectiveness of the prodrugs depends on the successful cleavage by the hydrolytic enzymes to release the active drugs for tumor killing.

### 3.2. Carbohydrate-Based Prodrugs for Specific Targeting

**Figure 4:**
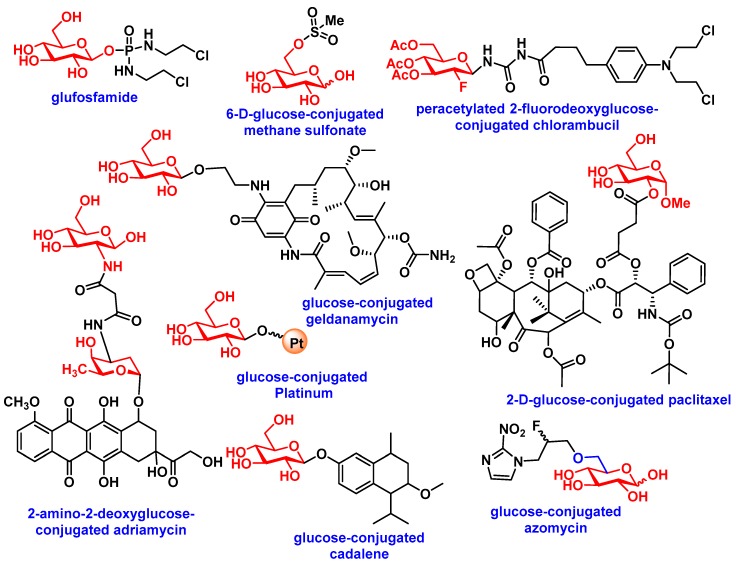
Glycoconjugated prodrugs for targeted delivery via GLUTs.

In 1995, Glufosfamide (Figure 4a), the first sugar-conjugated prodrug, was synthesized by Wiessler et al. in an attempt to decrease molecular toxicity and increase the cancer selectivity of a DNA alkylating aglycon ifosfamide mustard [50]. Glufosfamide showed 4.5-fold less toxicity than its active aglycon ifosfamide in rats (in mg kg^−1^) [50]. The first human clinical trial, with 20 patients, was initiated in Europe by Briasoulis et al. in 1997 [51]. More clinical trials ensued and further patients with various solid tumors were evaluated in Japan and USA; results proved promising [11]. Many anticancer drugs, such as chlorambucil, busulfan, docetaxel, and paclitaxel, were conjugated with several monosaccharides (see Figure 4), utilizing varying linkers, such as esters, amides, ureas, and succinic acids. Several groups reported preliminary biological assessments of the library of glycoconjugated derivatives by comparing the derivatives with their respective aglycons. A recent review by Calvaresi et al. discussed the glycoconjugation of some active cancer therapeutics [11]. Table 1 highlights some recent glycoconjugated anticancer agents, with their biological activity and mode of delivery towards cancer cells via glucose transporters (also see a comprehensive review by Calvaresi) [11].

Carbohydrate-based polymers, such as hydrogels, nanoparticles, micelles, and nanogels, have been shown to be promising delivery vehicles. Hydrophilic or hydrophobic drugs loaded onto hydrogels, through noncovalent interactions, allow in-situ release under specific and well-regulated conditions. Blanchette et al. synthesized P(MAA-g-EG) hydrogel nanoparticles to investigate the oral delivery of hydrophilic anticancer drug bleomycin [52]. This co-polymer solution mixture consists of methacrylic acid (MAA), a hydrophilic monomer, and ethylene glycol (EG) in a 1:1 molar ratio. To allow in situ polymerization, bleomycin was added to the above solution and then treated with UV light to initiate free radical polymerization, forming hydrogel nanospheres P(MAA-g-EG) [52]. For the oral delivery of hydrophobic drugs, Puranik et al. used hydrophobic monomers, such as *tert*-butyl methacrylate (*t*-BMA), *n*-butyl methacrylate (nBMA), *n*-butyl acrylate (nBA), and methyl methacrylate (MMA), along with MAA [53].

To improve the bioactivity and biodegradability of nanoparticles (NPs), and to reduce side effects, scientists are currently trying to find polysaccharide-based NPs (PNPs) for many cancer related treatments. Different polysaccharides, such as chitosan, hyaluronic acid (HA), chondroitin sulfate, heparin, alginate, and pullulan, have been used for the development of various NPs [54]. Furthermore, for specific delivery to the colon, different types of polysaccharides, such as chitosan and cyclodextrin, chitosan and pectin, have been utilized for achieving colon-specific delivery [54]. A number of anti-cancer drugs, such as doxorubicin (DOX), paclitaxel, docetaxel, cisplatin, and 5-fluorouracil, have been conjugated to polysaccharides to achieve their polysaccharide-based delivery to cancer cells [55]. 

Recently, a pH sensitive DOX-loaded mesoporous silica nanoparticle (MSN) was conjugated with lectin for the treatment of bone cancer [56]. The building blocks of the multifunctional nanosystem consisted of a polyacrylic acid (PAA) capping layer, which is grafted to MSN, a glycan (sialic acid) targeting ligand, and plant lectin concanavalin A (ConA). This nano-device exhibited 8-fold higher toxicity on tumor cells than free DOX.

## 4. Iminosugar Analogs for Cancer Therapy

### 4.1. Aberrant N-Linked Glycosylation and Inhibition of Glycosidase Enzyme

**Figure 5:**
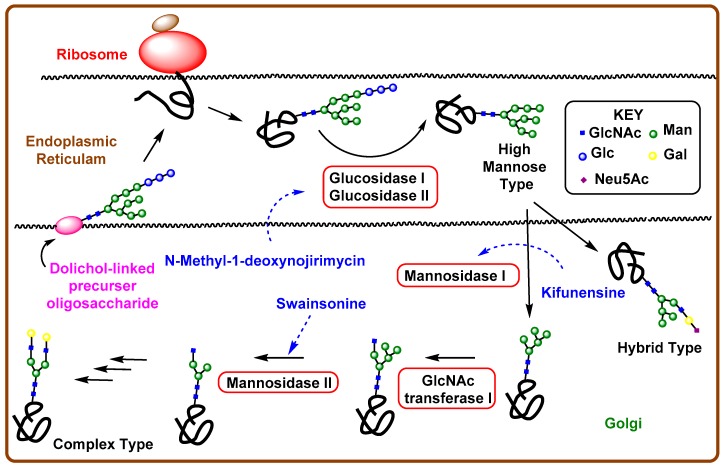
Biosynthetic pathways for *N*-glycans and iminosugars, inhibiting different glycosidase enzymes.

Cell glycans are synthesized by many glycosyltransferases, and the process generally occurs in the endoplasmic reticulum or Golgi apparatus [70,71]. For example, some enzymes are responsible for the aberrant addition or truncation of the carbohydrate moieties on cell surface lipids, proteins, or peptides, which also play roles in distinguishing tumor cells from normal cells and tumor metastasis [72]. Similar to mutations in genes, due to aberrant *O*-glycosylation, *N*-linked oligosaccharides also play a critical role in tumor cell progression and mitosis. The biosynthesis of *N*-glycoproteins, that normally begins in the endoplasmic reticulum (ER), involves three major sequences. The first phase involves the synthesis of a dolichol-linked precursor oligosaccharide, GLc_3_Man_9_GlcNAc_2_-PP-Dol. The lipid molecule, dolichol, is found to be attached to the membrane of ER and contains one phosphate group to add various sugar molecules (two GlcNAc, nine mannose, and three glucose), with a pyrophosphate group. After synthesis of the precursor molecule, it gets transferred to an asparagine (Asn) residue of a protein by oligosaccharyltransferases. The final phase involves modification of the oligosaccharide chain by several trimming reactions, such as the stepwise removal of three glucose molecules and up to six mannose residues by glucosidase I and II. Following the attack by enzymes, the remaining central core undergoes further processing reactions, e.g., the addition of other sugars, such as *N*-acetylglucosamine, galactose, neuraminic acid, *L*-fucose, and *N*-acetylgalactoseamine performed by several glycotransferase enzymes to produce three different types of *N*-glycans (Figure 5). High mannose-type glycans contain additional mannose units, whereas both hybrid and complex types contain additional monosaccharide units attached to the core. Due to the complex nature of those aglycons, any variation in this biosynthesis may lead to oncogenesis and tumor metastasis. Therefore, the identification of new therapeutic strategies for the inhibition of those glycosidases has captured the attention of scientists to prevent aberrant *N*-glycosylation and, hence, halt cancer.

### 4.2. Iminosugars as Enzyme Inhibitors

**Figure 6:**
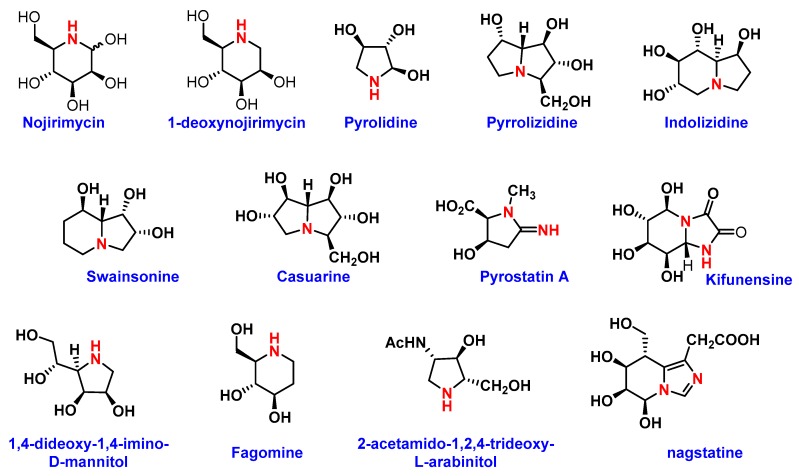
Structures of different iminosugars.

Several naturally-occurring iminosugars have noted anticancer activity through their ability to target the *N*-glycan biosynthesis pathway. These azo sugars are carbohydrate analogs, in which the oxygen heteroatom position in the ring is replaced by a more basic trivalent nitrogen. This simple substitution makes synthesis challenging and generates opportunities for evaluation of biological activities. The most common naturally occurring rings for this class are pyrrolidine, piperidine, pyrrolizidine, indolizidine, and nor-tropane (Figure 6). Since the first isolation of nojirimycin from *Streptomyces roseochromogenes* R-468, as an antibiotic, approximately 200 naturally occurring azo sugars have been isolated, but still very few are available for pharmaceutical applications [73]. The initial biological evaluations of these sugar analogs indicate their glycosidase and glycosyltransferase inhibitory properties [73]. Recently, these analogs have gained importance in the development of new anticancer drugs. Most of the investigations were, however, carried out on plant glycosidases. Hence, further work is required on mammalian glycosidases to uncover their potential in cancer research.

(−)-Swainsonine, (1*S*,2*R*,8*R*,8a*R*)-1,2,8-trihydroxyindolizidine is the most investigated iminofuranoside that can be found in several natural sources. It is an effective inhibitor of lysosomal *α*1-3 and *α*-1-6-mannosidase and Golgi *α*-manosidase II. The inhibition of Golgi α-manosidase II by (−)-swainsonine can block the expression of the *β*(1→6)-branched complex type *N*-glycans in malignant human and rodent cells. With this finding, a phase I study was conducted, in which the potency of glycoprotein processing iminosugars and swainsonine hydrochloride (GD0039) were tested as anti-cancer drugs. Subsequently, clinical phase II trials were conducted in Canada in and around 2002 [13]. The phase II results from 40 patients with advanced renal cell cancer or 5-florouracil (5-FU) resistant advanced colorectal cancer were deemed auspicious. Unfortunately, during the pharmacokinetics and pharmacodynamics investigations of oral GD0039, all the cancer patients at an advanced stage discontinued treatment due to disease progression or toxicity. However, GD0039 was shown to prevent metastasis, inhibit the growth of tumor cells, activate lymphocyte proliferation, and enhance T-cell stimulation. Inspired by biological interactions, various derivatives have been synthesized and anti-cancer activities evaluated, however none of them were observed to be as potent as their predecessor. On the other hand, casuarine, a pentahydroxylated pyrrolizidine, was found to be a good glucosidase inhibitor, showing immune responses such as increasing levels of cytokines IL-2, IL-12, and IFN- γ. Interestingly, those immunological responses do not necessarily depend on glucosidase inhibition. Other iminoalditols and their specific glycosidase inhibitory activities are listed in Table 2.

## 5. Carbohydrate-Based Diagnosis

**Figure 7:**
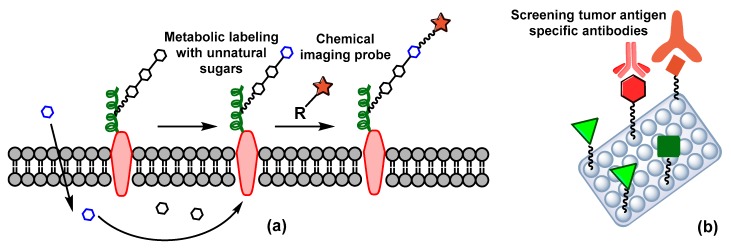
Early detection of cancer (**a**) metabolic oligosaccharide engineering (MOE) technology, (**b**) glycan micro array strategies.

Some serum glycoprotein biomarkers, such as carcino-embryonic antigen (CEA), carbohydrate antigens 19-9 (CA19-9) and 125 (CA125), alpha-fetoprotein (AFP), and prostate-specific antigen (PSA) have been found to be useful in the initial detection of colon, ovarian, and prostate cancers [79]. Alternatively, early detection is possible in positron emission tomography (PET), based on an increased concentration of 2-flurodeoxy-D-glucose (^18^FDG) in tumor cells. As cancer cells are more metabolically active, another imaging probe strategy, named metabolic oligosaccharide engineering (MOE) technology, has recently opened a new era in cancer diagnosis. In this strategy, non-natural derivatives of sialic acid, GalNAc, and fucose are supplied exogenously and get incorporated, using biosynthetic machinery, within the cellular glycans chains (Figure 7a). Those glycans get tagged with chemical imaging probes using biorthogonal reactions, and then are monitored with magnetic resonance imaging (MRI) [80]. 

The use of specific lectins to screen potential carbohydrate tumor biomarkers has gained traction in the diagnosis of cancer types with a lack of serum biomarkers. Lectins can bind with selective carbohydrates, are able to distinguish abnormal glycosylation, and trigger the mechanism required for tumor cell apoptosis [81]. A group of lectin proteins, such as *Amaranthus caudatus* agglutinin (ACA), *Artocarpus integrifolia* agglutinin (AIA), *Arachis hypogea* agglutinin (AHA), *Vicia villosa* lectin (VVL), *Griffonia simplicifolia* agglutinin I (GSA I), and *Ulex europaeus* agglutinin I (UEA I) can recognize the Tn, TF, and STn alteration of CA125 and human epididymis secretory protein 4 (HE4) antigens (Figure 7b) [82]. Similarly, glycan microarray strategies have been utilized to detect the presence of antibodies against specific antigens (e.g., Globo H) in cancer patients’ serum [15]. The array is composed of various carbohydrates on a solid support and provides high-throughput cancer related glycan–protein interactions (Figure 7b). 

## 6. Conclusion

Due to their ubiquitous nature, carbohydrates have long-been used as a means for cancer diagnosis and for the development of safe, small molecule therapeutics. Although several carbohydrate agents have successfully been synthesized and processed for clinical trials, the therapeutic responses have not lived up to their promise in treating cancer. Results from the use of commercially available carbohydrate-based therapeutic agents has also not been shown to be highly significant. Therefore, more exhaustive studies are required to explore potent therapeutic agents able to combat cancer. One approach might be the development of vaccines with non-natural synthetic antigens, which may overcome the immunosuppressive nature of carrier proteins. Another strategy might be an expansion on the co-administration of a vaccine and glycoconjugated prodrug, which can promote specific drug delivery as well as perturb cancer. Although iminosugars have proven to be glycosidase inhibiting agents for *N*-glycan biosynthesis, further evaluations are required for extended development toward novel drugs. 

## Figures and Tables

**Table 1:** List of glycoconjugated prodrugs.

Aglycons	Conjugated Sugars	Response of Glycoconjugates Compared to Aglycon in In-Vitro or In-Vivo	Transportation Mode	Ref(s)
Chlorambucil	Peracetylated 2-fluorodeoxyglucose	Human fibroblasts, MCF-7 (25-fold more active) and Mice (Increased in MTD)	-	[57]
Docetaxel	Glucose, galactose, mannose, xylose	B16 murine melanoma cells (3 to 18-fold more active)	-	[58]
Docetaxel	galactose	Syngeneic P388 murine leukemia tumor model (equivalent)	-	[59]
Paclitaxel	Glucose, glucuronic Acid	HUV-EC-C and CHO-K1, NCI-H838, Hep-3B, A498, MES-SA, HCT-116, NPC-TW01, MKN-45 (All less toxic)	Partially GLUT-1, /GLUT-3/GLUT-4 mediated	[48,60]
Chlorambucil	Amino derivatives of glucose, mannose, galactose, xylose, lyxose, D-threoside	NCI-H460, A549, Du145, SKOV3, Hep3b, SF268, MCF7, HT29, HCT15, H1299 (induce decrease in cell growth)	-	[61]
Benzylguanine	Glucose	HeLa S3 and HeLa MR cells (inhibition of *O*^6^-methyl-guanine-DNA methyltransferase, MGMT)	-	[62]
Azomycin	Glucose	Several immortalized murine and human cancer cells (improved selectivity towards hypoxic tumor as radiosensitizer)	GLUTs mediated	[63]
Adriamycin	2-amino-2-deoxy-glucose	MCF-7, Bel-7402, HepG2, MDA-MB-231, U87MG, HELF, SKOV3, and S180, HELF and mice (enhance selectivity towards cancer cells)	GLUTs mediated	[64]
Geldanamycin	Glucose, lactose, galactose	SW620, HT29, MCF7, K562 (one showed 3- to 40-fold enhanced activity with *β*-galactosidase)	-	[65]
Platinum	Glucose	DU145, RWPE2	GLUTs mediated	[66]
Cadalene	Glucose, lactose, galactose	In vitro (less toxic) and in vivo (reduced tumor size)	-	[67]
Ketoprofen	Glucose	Cross blood−brain barrier (BBB)	GLUTs mediated	[68]
Nordihydroguaiaretic acid	Galactose, glucose	NCI/ADR-RES, Hep3B, MCF-7, HT-29	-	[69]

**Table 2:** List of iminosugars and their inhibitory effects.

Amino Sugars	Glucosidase Inhibition	Other Anti-Tumor Activities	Ref (s)
Swainsonine	Lysosomal α-1-3- (IC_50_ 0.70 nM) and α-1-6-mannosidase (K*_i_* 40 nM) and Golgi α-mannosidase	Inhibits growth of tumor cells	[13]
1,4-Dideoxy-1,4-imino-D-mannitol	α-mannosidase, Lysosomal Golgi α-mannosidase II, glycogen phosphorylase	Human Glioblastoma and Melanoma Cells	[74]
1-Deoxymannojirimycin	α-1-2-mannosidase (IC_50_ 0.02 mM), Golgi α-mannosidase II (IC_50_ 400 µM)	Interact with recombinant tumor necrosis factor (rTNF) and recombinant interleukin 1 (rIL-1)	[75]
2-aminomethyl-5-(hydroxymethyl) pyrrolidine3,4-diol derivative	Jack bean α-Mannosidase (IC_50_ 55 µM)	Inhibits growth of human glioblastoma cells and melanoma cells, DNA, synthesis of proteins	[74,76]
Castanospermine	α- and β-glucosidases	Inhibitor of breast cancer	[77]
1-deoxynojirimycin	Glucosidase I and II	Anti-metastatic activity, reduce adhesion of tumor cells to vascular endothelium, inhibit cellular transformation, prevent morphological differentiation of endothelial cells	[13]
(+)-Lentiginosine	amyloglucosidases	Inhibits ATPase and Chaperone Activity of Hsp90	[78]
Siastatin B	β-glucuronidase, NAG-ase	Antimetastatic activity	[13]
